# Macrophages and Phospholipases at the Intersection between Inflammation and the Pathogenesis of HIV-1 Infection

**DOI:** 10.3390/ijms18071390

**Published:** 2017-06-29

**Authors:** Francesca Spadaro, Serena Cecchetti, Laura Fantuzzi

**Affiliations:** 1 Confocal Microscopy Unit, Core Facilities, Istituto Superiore di Sanità, Viale Regina Elena 299, 00161 Rome, Italy; 2 Center for Global Health, Istituto Superiore di Sanità, Viale Regina Elena 299, 00161 Rome, Italy

**Keywords:** monocyte/macrophage, phospholipases, HIV-1, inflammation

## Abstract

Persistent low grade immune activation and chronic inflammation are nowadays considered main driving forces of the progressive immunologic failure in effective antiretroviral therapy treated HIV-1 infected individuals. Among the factors contributing to this phenomenon, microbial translocation has emerged as a key driver of persistent immune activation. Indeed, the rapid depletion of gastrointestinal CD4^+^ T lymphocytes occurring during the early phases of infection leads to a deterioration of the gut epithelium followed by the translocation of microbial products into the systemic circulation and the subsequent activation of innate immunity. In this context, monocytes/macrophages are increasingly recognized as an important source of inflammation, linked to HIV-1 disease progression and to non-AIDS complications, such as cardiovascular disease and neurocognitive decline, which are currently main challenges in treated patients. Lipid signaling plays a central role in modulating monocyte/macrophage activation, immune functions and inflammatory responses. Phospholipase-mediated phospholipid hydrolysis leads to the production of lipid mediators or second messengers that affect signal transduction, thus regulating a variety of physiologic and pathophysiologic processes. In this review, we discuss the contribution of phospholipases to monocyte/macrophage activation in the context of HIV-1 infection, focusing on their involvement in virus-associated chronic inflammation and co-morbidities.

## 1. Introduction

More than 30 years after its discovery in the early 1980s, infection with human immunodeficiency virus type 1 (HIV-1) and acquired immunodeficiency syndrome (AIDS) still represent a challenging health problem worldwide. The introduction of highly active antiretroviral therapy (HAART) in the mid-1990s resulted in a drastic reduction of morbidity and mortality. However, despite the extraordinary success of HAART to increase the life expectancy of HIV-1-infected individuals, several factors hinder the achievement of a cure. In particular, although current therapy strongly inhibits HIV-1 replication, it does not eradicate the virus. In fact, most of the patients on effective therapy have low and stable residual plasma viremia, which represents the main obstacle to a cure [1,2]. This residual viremia is linked to the persistence of viruses in cellular and anatomical reservoirs in the body during therapy. The establishment of a small pool of long-lived latently infected cells early in infection provides the virus with a cellular niche that ensures its maintenance for decades during therapy. In addition, a suboptimal penetration of many antiretroviral drugs in anatomical sites such as the central nervous system (CNS) contributes to low-rate viral replication and release from viral reservoirs. Finally, even with long-term effective therapy, HIV-1-infected persons have residual levels of inflammation and immune activation, which are strongly associated with the magnitude of the viral reservoir [3]. This suggests that HIV-1 persistence and residual inflammation are interdependent and represent the major challenges to achieve a cure.

In the last decade, the phenomenon of microbial translocation was widely recognized as the principal driver of immune activation, inflammation, and HIV-1 disease progression [4,5]. In the early stages of infection, HIV-1 replicates in gut-associated lymphoid tissues, resulting in a rapid and massive depletion of mucosal CD4^+^ T lymphocytes. This event is followed by gut epithelium damage and increased translocation of microbes and microbial products, such as lipopolysaccharide (LPS), from the gut lumen to portal and systemic circulation, ultimately provoking a persistent, systemic inflammation. The growing amount of data sustaining this concept has deeply changed the view of HIV-1 infection, which is now also considered as a chronic inflammatory disease. Indeed, long-term-HAART-treated HIV-1-infected individuals exhibit a heightened risk for several serious non-AIDS degenerative diseases (i.e., cardiovascular and neurocognitive diseases, non-HIV associated cancers, liver, bone and kidney dysfunctions, and other end-organ illnesses), which depict a pattern of accelerated aging and result in reduced life expectancy. Nowadays, these morbidities represent the major cause of mortality in virologically suppressed individuals and are considered a direct or indirect outcome of chronic inflammation [3]. Hence, it is crucial to better understand the causes and develop interventions to attenuate the effects of immune activation and inflammation in HIV-1-infected people.

Persistent activation of innate immune cells, above all monocytes and macrophages, is thought to represent one of the major drivers of chronic inflammation and co-morbidities in HIV-1-infected individuals. This is supported by recent works demonstrating that plasma markers of innate immune activation, including IL-6 and soluble TNF-α receptor, predict non-AIDS morbidity and mortality more strongly than cellular markers of T cell activation [6,7]. In addition, high plasma levels of soluble CD14 (sCD14), which is shed by monocytes/macrophages in response to LPS and other inflammatory stimuli, are associated with an increased risk of all-cause mortality in HIV-1-infected subjects [8]. Very recently, plasma soluble CD163 (sCD163), a more specific marker of monocyte/macrophage activation that is shed in the course of the pro-inflammatory response, was identified as an independent marker of all-cause mortality in HIV-1^+^ individuals [9].

Several signal transduction pathways are involved in monocyte/macrophage activation by HIV-1 as well as microbial products, such as LPS [10,11]. Among these pathways, phospholipase (PL) signaling is increasingly recognized to be essential in the orchestration of the inflammatory response [12]. These enzymes catalyze phospholipid cleavage, leading to the production of lipid mediators or second messengers that have central roles in signal transduction. In this review we discuss the contribution of PLs, above all PLC and PLA_2_ families, to monocyte/macrophage differentiation/activation and chronic inflammation and their involvement in HIV-1 infection-associated microbial translocation and co-morbidities, mainly focusing on cardiovascular and neurological diseases.

## 2. Macrophage and Phospholipase Biology

### 2.1. Phospholipases

Growing evidence is accumulating on the importance of PLs as key players in HIV-1 infection and host immune-metabolic dysfunctions. PLs hydrolyze phospholipids into fatty acids (FAs) and other lipophilic substances. They are classified into four distinct families, named PLA, PLB, PLC and PLD, based on the stereospecifically numbered sites within phospholipids where they promote cleavage (for and extensive review see [12]). Phospholipid hydrolysis is a widespread response triggered by most cytokines, growth factors, neurotransmitters, hormones, and other extracellular signals, and its products include many of the most important second messengers, such as inositol 1,4,5-trisphosphate (IP_3_) and diacylglycerol (DAG), that are implicated in cellular responses. Major substrates of catalytically active PLs are the glycerophospholipids phosphoinositides (PIs) and phosphatidylcholines (PCs). However, these enzymes can also act on other phospholipids, such as phosphatidylethanolamine, sphingomyelin, lysophosphatidylcholine (LPC) and lysophosphatidylinositol [13]. The PC cycle generates second messengers, growth factors, mitogens, and mediators of inflammation, such as DAG, phosphatidic acid, lysophosphatidate, LPC, arachidonic acid (AA), glycerophosphate, and free FAs (FFAs), through the action of different stimulated PL activities (i.e., PLA_2_, PC-PLC and PLD) [14]. AA is a signaling molecule or, upon downstream modification by various enzymes (mainly cyclooxygenases and lipoxygenases), it is modified into eicosanoids, including prostaglandins (PGs) and leukotrienes (LTs), which act in the body as the “AA cascade” and play a relevant role in inflammation [12,15]. In this review we will focus on cytosolic PLA_2_s (cPLA_2_ and iPLA_2_) isoforms, lipoprotein-associated PLA_2_s (Lp-PLA_2_s, or platelet-activating factor-acetylhydrolases PAF-AHs, or groups VII and VIII PLA_2_s), different groups of secreted PLA_2_s (sPLA_2_s) and PC-PLC [12]. PIs are inositol-containing phospholipids comprising phosphatidylinositol and its phosphorylated derivatives. They have a specific function correlated to distinct membrane compartments and exert a key role in signaling, trafficking and actin cytoskeletal dynamics [16]. PI-PLC isozymes comprise a related group of proteins that cleave the polar head group from PIs, releasing IP_3_ and DAG [12,17].

### 2.2. Role of Phospholipases in Regulating Differentiation, Polarization and Immune Functions of Monocytes/Macrophages

Macrophages are myeloid lineage cells of the innate immune system that play critical roles in several processes, as extensively described in previous reviews [18,19]. They sense and eliminate invading microorganisms and orchestrate the development of innate and adaptive immune responses to pathogens. Macrophages express on their surface specific pattern recognition receptors (PRRs), which recognize pathogen associated molecular patterns, such as LPS. This interaction triggers intracellular signaling leading to the expression of a complex network of cytokines and chemokines, which together coordinate the early host response to infection as well as activate and shape the adaptive immune response [5].

Lipid signaling is increasingly recognized to play a crucial role in macrophage biology by affecting multiple cellular pathways. In the context of macrophage differentiation, PI-PLCγ2 was recently described as an important mediator of myeloid lineage commitment. Indeed, it promotes macrophage differentiation when associated with ERK1/2 and NFAT activation in monocytes stimulated with macrophage colony-stimulating factor (M-CSF), whereas it supports granulocytic lineage commitment when coupled to Jak1/Stat3 activation [20]. The involvement of PI-PLCγ isozymes in Fcγ receptor-dependent phagocytosis in macrophages was investigated in several studies. Phosphorylated PI-PLCγ1 concentrates early at nascent phagosomes, supporting PKCε translocation and uptake of IgG-opsonized targets [21]. Even before receptor engagement, macrophages constantly probe their surroundings for particulate or soluble antigens by emitting actin-rich protrusions. This constitutive membrane ruffling is independent from PI-PLCγ1 and PI-PLCγ2 activation, but requires the concerted action of PI-PLCβ1, PI-PLCβ3 and DAG kinase [22].

Two major activation states of macrophages, strictly related to environmental factors, were described: M1 classically activated cells, which produce high levels of pro-inflammatory cytokines and mediate resistance to pathogens, and M2 alternatively activated macrophages, which are involved in parasite control and immune regulation [23]. This system is finely controlled by several endogenous molecules that interact in a dynamic complexity to limit the magnitude and duration of the inflammatory response, thus avoiding immunopathology. PLs and their lipid messengers contribute to the signal transduction pathways involved in macrophage polarization. In particular, PI-PLCβ2 and PI-PLCβ3 isozymes play a crucial role in inhibiting the switch from M1 to M2 phenotype [24,25], and lipids generated by iPLA_2_β favor M1 polarization [26]. In addition, differently polarized macrophages were shown to own peculiar panels of expression and intracellular localization of PI-PLC enzymes, which slightly change following triggering with inflammatory stimuli [27].

Interestingly, PL activity deeply affects the complex network of cytokines and chemokines released by macrophages. An important contribution is given by AA metabolites (mainly PGE_2_), which critically regulate the release of several cytokines (e.g., TNF-α, IL-12 and IL-10) and chemokines (e.g., CCL2, CXCL8, CCL3, CCL4, and CXCL10) from these cells [28,29]. Among the pro-inflammatory cytokines produced by macrophages, IL-1β is a good example of how different PLs act coordinately to control cytokine release. Indeed, whereas iPLA_2_ is involved in the processing of the IL-1β inactive precursor, PC-PLC and cPLA_2_ are required for lysosome exocytosis and IL-1β secretion [30]. iPLA_2_ also promotes macrophage proliferation [31], spreading and adhesion during inflammation [32], phagocytosis [33] and chemotaxis in response to CCL2 coupled to cPLA_2_ activity [34]. Macrophage adhesion, spreading and migration, as well as the respiratory burst and the inflammatory responses induced by Toll-like receptor (TLR) signaling are also controlled by the products of PI-PLCγ activity [35].

### 2.3. A Loop Intersecting Phospholipases, Macrophages and Chronic Inflammation

There is an emerging consensus on the central role of macrophages, particularly M1-like cells, in triggering the uncontrolled immune responses that characterize diseases with inflammatory pathogenesis [36]. Interestingly, the release of pro-inflammatory cytokines, such as TNF-α, IL-6 and CCL2, indicators of the M1 phenotype, strictly depends on PL signaling, and in turn the same cytokines can stimulate PL activation in a network that amplifies inflammation. In particular, PC-PLC in macrophages is critically required for the expression of TNF-α, IL-6 and IL-10, as well as for IL-1β secretion, in response to inflammatory stimuli [30,37,38]. TNF-α and IL-1β in turn stimulate PC-PLC activity in a positive feedback loop, leading to DAG production and NF-κB activation, which can induce TNF-α itself, as well as other inflammatory cytokines, such as IL-6 and CCL2. To further ensure a signaling that supports a persistent inflammatory state, both TNF-α and IL-1β can also stimulate a neutral sphingomyelinase that leads to PLA_2_ activation and PGE_2_ and LT production [39].

Given the central role of eicosanoids and their related bioactive lipid mediators in the onset and resolution of inflammation [40], several studies focused on PLA_2_ family as potential pharmacological target for the control of immune disorders and inflammatory responses [15,41]. In particular, it was suggested that iPLA_2_ activity drives the initial phase of the inflammatory response through synthesis of PGE_2_, LTB_4_ and IL-1β, whereas sPLA_2_ and cPLA_2_ are activated during the resolution phase [42]. The same enzyme can sometimes be linked to both the initiation and resolution of inflammation. For example, TLR4-mediated cPLA_2_α activation in macrophages leads to AA release, synthesis of PGE_2_ and accumulation of a lipoxin precursor, which is subsequently hydrolyzed by cPLA_2_α itself and converted to bioactive lipoxins by 5-lypoxygenase, in order to inhibit additional leukocyte recruitment during inflammatory resolution [40].

Among the chemoattractants released by damaged tissues, CCL2 plays a major role in selectively recruiting monocytes/macrophages in response to inflammatory stimuli. In this process, besides the key role of PLD activity [43], iPLA_2_β and cPLA_2_α are required and act in parallel to provide distinct lipid mediators at different intracellular sites, affecting monocyte directional migration and speed [34]. The recruitment of monocytes to the sub-endothelial space of the blood vessel wall is a critical step in atherosclerosis, a progressive vascular disease whose prevention and treatment are becoming urgent topics for the general population [44] as well as for HIV-1 infected persons, which are particularly prone to early atherosclerosis [45]. This disease is an excellent example of chronic inflammatory disorder in which different PLs contribute to the pathologic process and macrophages are essential in triggering and maintaining the inflammatory response during all stages of atherogenesis [46]. Of note, mice genetically deficient for CCL2 or its receptor CCR2 are protected from vascular lesions in a number of atherosclerosis models [47].

**Figure 1:**
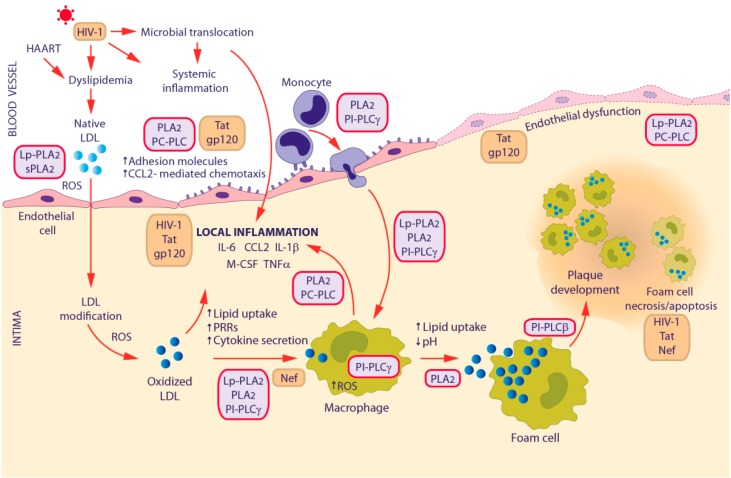
Schematic representation of the involvement of inflammation, phospholipases and HIV-1 infection in the pathogenesis of atherosclerosis. The figure depicts the major pathogenic events involved in atherosclerosis progression. Key sequential steps are: (i) accumulation and oxidation of LDL by ROS within the artery wall; (ii) recruitment/infiltration of monocytes and their differentiation into macrophages, which become foam cells on uptake of oxidized LDL; and (iii) foam cell coalescence into a lipid necrotic core leading to plaque development. Local chronic inflammation is the major trigger of atherogenesis. Light purple and orange boxes represent PL and HIV-1 contribution to these processes, respectively. CCL2, C-C motif chemokine ligand 2; HAART, highly active antiretroviral therapy; IL, interleukin; LDL, low-density lipoprotein; M-CSF, macrophage colony-stimulating factor; PRRs, pattern recognition receptors; ROS, reactive oxygen species; TNF-α, tumor necrosis factor α.

Along with the cardiometabolic risk factors, chronic inflammation is considered as a predominant driving force of atherogenesis [48,49]. In this context, PLs have attracted attention as potential therapeutic targets in virtue of their critical pro-inflammatory properties [44,50,51], which may be linked, at least in part, to their crucial role in macrophage activation. The key steps of the atherosclerotic process are represented in Figure 1 and have been extensively described in previous reviews [48,49]. The atherosclerotic intima-plaque milieu is complex and lipid mediators are well recognized to activate the inflammasome and PRRs (such as CD14, CD36, TLRs and low-density lipoprotein (LDL) binding protein) to induce both pro- and anti-inflammatory M1 and M2 macrophage subpopulations. Recent advances indicate that additional plaque-specific macrophage phenotypes can co-exist in the plaque and suggest that lesion progression is more related to the macrophage phenotype than to the absolute number of these cells within the plaque [52].

LDL is the lipoprotein generally linked to atherosclerosis. A key component of LDL is LPC, whose pro-inflammatory properties are mediated via PAF receptor [53]. LPC stimulates the production of reactive oxygen species (ROS), such as superoxide, by endothelial cells [54]. In addition, PI-PLCγ1, activated by TLR4 stimulation with minimally oxidized LDL, induces ROS generation in macrophages, leading to the production of pro-inflammatory cytokines such as TNF-α, IL-1β and IL-6 [55], all having central roles in the pathogenesis of atherosclerosis [49].

In monocytes, LPC initiates two parallel signaling pathways, sequentially activating PLD and cPLA_2_ in the first, and cPLA_2_ concomitantly with sPLA_2_ in the second, all leading to AA release [53]. Of note, oxidized FFAs stimulate the expression of adhesion molecules on endothelial cells, induce chemotaxis of monocytes and promote their entry in the sub-intima space of blood vessel walls [54]. In addition, FFAs may contribute to acidification of atherosclerotic lesions, which are often hypoxic and acidic as a consequence of the abundant accumulation of lipid-scavenging macrophages. In turn, the acidic extracellular pH affects important macrophage functions by enhancing FcγR-mediated phagocytosis and triggering IL-1β and IL-18 secretion [46]. Several sPLA_2_ enzymes are present in human atherosclerotic arteries, including sPLA_2_-V that is more active against LDL at acidic pH, contributing to modification and aggregation of LDL [46]. More generally, sPLA_2_ (groups IIA, V and X) have potential pro-atherogenic features derived from lipoprotein remodeling, activation of inflammatory pathways, binding to integrins (to induce proliferative signals mediated by ERK 1/2) and increased expression of adhesion molecules on endothelial cells, including ICAM-1 and VCAM-1 [56]. These molecules, along with E- and P-selectins and carotid intima-media thickness, are considered useful parameters of vascular endothelial dysfunction and early atherosclerosis, whereas sPLA_2_-IIA was proposed as a biomarker to assess the prognostic impact of inflammation on the risk of coronary artery disease in healthy individuals [57]. Although Lp-PLA_2_ becomes progressively activated as atherosclerosis progress, its contribution may depend on the type of lipoprotein particle, LDL or high-density lipoprotein, with which it is associated in the bloodstream [50,54]. Lp-PLA_2_ is mainly produced by monocytes/macrophages and is released in the circulation when inflammatory stimuli induce monocyte differentiation to macrophage [58]. This enzyme hydrolyzes the sn-2 acyl-chain of the phospholipid substrate on LDL surface releasing LPC and oxidized FFA, which concentrate in the sub-intimal space contributing to the development of foam cells and plaque lipid core [59]. In virtue of the important pro-atherogenic features of these metabolites, the measurement of Lp-PLA_2_ activity was included among the biomarkers used in clinical practice for the stratification of adult asymptomatic patients at intermediate or high cardiovascular risk [54], and was recently proposed as a new marker to identify early atherosclerotic changes in hypercholesterolemic dyslipidemic children [60].

Besides PLA_2_ enzymes, also PC-PLC signaling plays a key role in atherosclerosis. Its crucial function in regulating NF-κB-dependent transcription of CCL2 in response to inflammatory agents (e.g., TNF-α, IL-1β, LPS, and oxidized LDL) in monocytes/macrophages, endothelial and smooth muscle cells is well known [61,62]. In addition, PC-PLC contributes to vascular endothelial cell dysfunction and progression of atherosclerosis through induction of apoptosis [62]. A critical involvement in atherosclerosis development was also uncovered for the PI-PLCβ family. Indeed, macrophage survival at sites of arterial lesions is strictly dependent on PI-PLCβ3 activity, which regulates the sensitivity to multiple inducers of apoptosis via PKC-dependent up-regulation of Bcl-XL [51].

In conclusion, inflammatory processes are firmly established to be central in the development and complications of atherosclerosis. Especially in the context of HIV-1 infection where monocytes are chronically activated, understanding the causes of the premature onset of atherosclerosis may lead to improved prevention and intervention strategies in this vulnerable population. Although targeting of various inflammatory pathways in experimental models of atherosclerosis was demonstrated to be beneficial in attenuating arterial injury and reducing disease progression, clinical translation has been disappointing thus far, since a clear efficacy has not yet been shown for any single agent [49].

## 3. Macrophage and Phospholipase Contribution to Immune Activation and Inflammation in HIV-1 Infection

Along with CD4^+^ T lymphocytes, macrophage lineage cells play key roles in HIV-1 pathogenesis throughout the course of infection [63,64]. Circulating monocytes can be categorized in three main subpopulations on the basis of CD14 and CD16 surface expression: classical (CD14^++^CD16^−^), intermediate (CD14^++^CD16^+^), and non-classical (CD14^+^CD16^++^) monocytes. Classical monocytes, which express CCR2 and migrate to inflammatory sites in response to CCL2, are resistant to HIV-1 infection, whereas CD16^+^ monocytes are highly susceptible to infection [65]. Interestingly, whereas the CD16^+^ subpopulation makes up only 5–10% of all peripheral blood monocytes, this percentage can increase up to ∼40% in HIV-1^+^ individuals. Furthermore, a novel subset of monocytes was identified during HIV-1 and simian immunodeficiency virus (SIV) infection. These cells express similar levels of CD14 and CD16 but lower CCR2, activation markers and inflammatory cytokines than classical monocytes, show defective phagocytosis and chemotaxis, and are refractory to viral infection [66]. Macrophage susceptibility to HIV-1 infection is dependent on tissue localization. Interestingly, M1 or M2 polarization leads to a restriction of viral replication in comparison to unpolarized cells [67]. Macrophage polarization was proposed as a possible connection between HIV-1 infection and immune suppression. Indeed, M-CSF may foster and/or prolong M2 activation of macrophages and microglia in brains of SIV-infected animals with encephalitis [68], which could contribute to CNS disease and other co-morbidities, as well as to immune dysfunction and polarization of immune responses toward immune suppression.

In addition to resting memory CD4^+^ T lymphocytes, macrophages may also participate in the establishment of tissue reservoirs in HIV-1 and SIV infection. Interestingly, detectable levels of infected macrophages were found in brain tissues of HAART-treated SIV-infected macaques [69]. During early stages of infection in these animals, a transient SIV latency was observed in macrophages/microglia [70], and more recently unexpressed HIV-1 DNA was also detected in macrophages/microglia in brain tissue from patients died with pre-symptomatic HIV-1 infection [71]. Paradoxically, although HAART has provided important results by decreasing CD4^+^ T cell depletion, it has exposed the core lentiviral nature of HIV-1 as a pathogen adapted to survive and cause slow progressive disease through colonization of macrophages, particularly in the CNS and in the gastrointestinal tract.

Besides their role in HIV-1 spread and persistence, infection of macrophages may directly promote disease, the principal mechanisms being alterations of macrophage functions and activation of inflammatory processes. Monocytes/macrophages isolated from HIV-1^+^ individuals show impaired migratory responses and reduced phagocytic activity, which result in an inefficient control of opportunistic infections and further enhancement of immune activation and disease pathogenesis. For example, alveolar macrophages from untreated- and HAART-treated patients have defective phagocytic activity, resulting in increased susceptibility to infections of the lower respiratory tract and respiratory dysfunctions [72].

Circulating monocytes and tissue macrophages represent an important source of pro-inflammatory cytokines and chemokines, which are associated with cardiovascular disease (CVD), HIV-1-associated neurological impairment and innate immune aging. Among these factors, CCL2 may play a critical role in HIV-1 pathogenesis. Although this chemokine can be produced by many cell types, monocytes/macrophages are the major source of CCL2 [73]. Its expression is increased during monocyte to macrophage differentiation [74] as well as in the course of HIV-1 infection, as demonstrated by several in vitro and in vivo studies [75]. In particular, either infection itself or exposure to viral proteins can induce an increase of CCL2 production in monocyte-derived macrophages (MDMs) [76,77]. In these cells, gp120-elicited production of CCL2 is mediated by PLCs, as demonstrated by previous studies conducted by our group [78,79]. Indeed, gp120 interaction with CCR5 determines a modification of the subcellular distribution and an increase of the enzymatic activity of PC-PLC. In turn, this enzyme is required for NF-κB activation, which subsequently leads to CCL2 production [78]. Furthermore, our work identified ERK-1/2 and nuclear PI-PLCβ1 as key intermediates in the PC-PLC-driven CCL2 secretion in MDMs [79]. All together, these results uncovered a concerted gp120-mediated signaling involving PLCs as a required step for HIV-1-induced expression of CCL2 in macrophages. This chemokine not only represents a major inflammatory mediator contributing to a plethora of HIV-1-related morbidities, but also acts as a cellular factor exploited by the virus to enhance its own replication [75,76,80]. Thus, PL-mediated signal transduction pathways may be relevant for the modulation of viral replication, at least in macrophages, and, in general, for the pathogenesis of HIV-1 infection. Interestingly, exogenous PC-PLC-triggered PC breakdown was shown to activate NF-κB and increase HIV-1 replication in chronically infected monocytes and T lymphocytes, thus suggesting that a cellular transduction pathway dependent on PC-PLC-mediated PC hydrolysis may play a key role in stimulating HIV-1 replication in these cells [81].

### 3.1. Microbial Translocation in HIV-1 Infection

**Figure 2:**
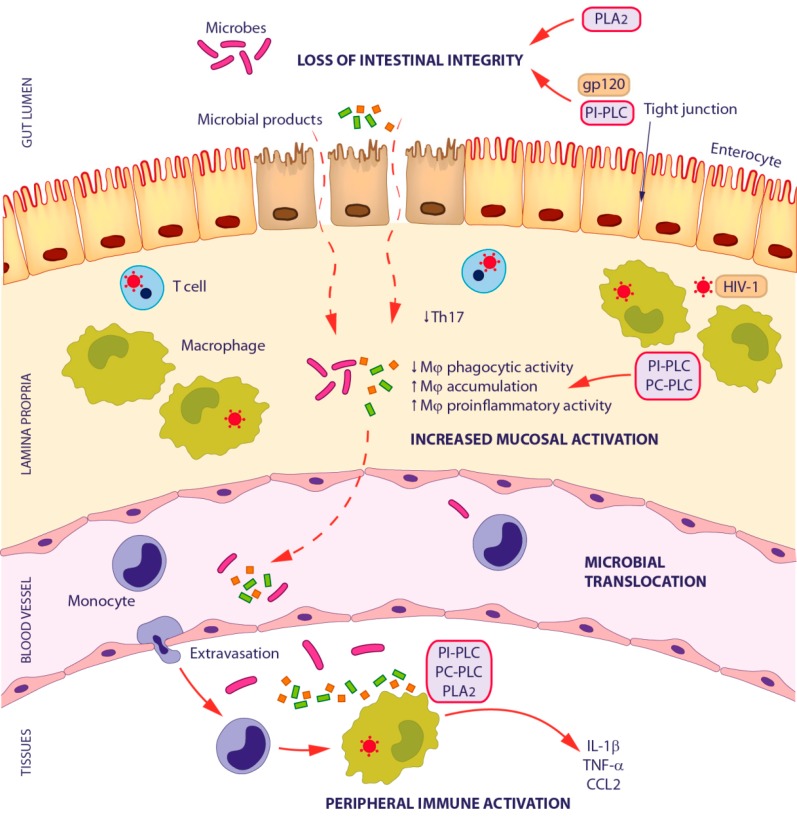
Schematic model of the contribution of phospholipases in the pathogenesis of microbial translocation in HIV-1 infection. Multiple mechanisms account for the loss of intestinal integrity in HIV-1 infection (enterocyte apoptosis, loss of tight junctions, local immune activation, depletion of Th17 cells, and macrophage dysfunction). Consequently, microbial products and pathogenic bacteria pass from the gut lumen to the lamina propria, then to the systemic circulation and finally to peripheral tissues. At these sites, microbes and their products further intensify local immune activation, which may be an underlying cause of disease progression and co-morbidities, such as neurological impairment and cardiovascular diseases. Light purple and orange boxes represent PL and HIV-1 contribution to these processes, respectively. Th17, T helper 17; Mϕ, macrophage; IL-1, interleukin-1; TNF-α, tumor necrosis factor-α; CCL2, CC chemokine ligand 2.

HIV-1-related microbial translocation is caused by a sequence of immunopathological events taking place at the gastrointestinal tract mucosa (Figure 2), which have been extensively described in previous reviews [4,5]. An important factor implicated in the pathogenesis of this condition is the accumulation in the gut mucosa of pro-inflammatory macrophages with impaired phagocytic activity. These cells may contribute to tissue injury induced by local inflammation without increasing their ability to eliminate luminal products that cross the damaged epithelial layer. Interestingly, the CCL2-CCR2 axis is involved in mucosal infiltration by macrophages, as a consequence of increased CCR2 expression on integrin β7–expressing monocytes of HIV-1-infected patients and improved mucosal secretion of CCL2 [82].

In some aspects, HIV-1-associated microbial translocation may resemble that observed in patients with inflammatory bowel disease. Intriguingly, the activity of PLA_2_ is increased in the intestinal epithelia of these patients. Furthermore, LPC stimulates microbial translocation in an in vitro enterocyte cell-culture model [83]. This suggests that in vivo conditions in which LPC and/or PLA_2_ levels are elevated at the mucosal epithelial surface or within the gut lumen may contribute to alter intestinal barrier function and increase epithelial permeability, thus favoring microbial translocation. The permeability of the intestinal epithelium may also be affected by gp120. In fact, this viral protein was shown to interact with Bob/GPR15, which is abundantly expressed on the basolateral surface of small intestinal epithelium, thus inducing Ca^2+^ signaling, microtubule loss and physiological changes resembling HIV-1-mediated enteropathy. Interestingly, these effects were inhibited by the PI-PLC inhibitor U73122 [84].

Although the mechanisms by which microbial translocation lead to immune activation are still controversial, a key pathogenic event is the triggering of innate immunity through PRRs. The unchecked continuous passage of bacteria and microbial components from the intestinal lumen to the systemic circulation stimulates innate immune cells, primarily monocytes and macrophages, through TLRs and other innate immune receptors, thus promoting systemic immune activation and the pro-inflammatory cytokine milieu associated with chronic HIV-1 infection. In keeping with this, it was recently reported that monocytes/macrophages from patients undergoing HAART are preferentially activated by circulating bacterial products [85].

Microbial translocation can be quantified either directly by measuring bacterial by-products in plasma (e.g., LPS and bacterial 16S ribosomal DNA or RNA fragments), or indirectly by sCD14, LPS binding protein, endotoxin core antibodies, and flagellin-specific IgG determination. LPS, a component of the Gram-negative bacterial cell wall acting as a TLR4 agonist, has been thus far considered a major marker of microbial translocation. Its interaction with CD14 expressed on monocytes/macrophages triggers NF-κB activation and cytokine production, thus leading to the systemic inflammatory response. The contribution of PLs to these pathways has long been recognized. In particular, PC-PLC signaling was identified as a crucial component of the CD14/LPS-mediated macrophage activation and inflammatory response [37]. In human alveolar macrophages, this enzyme mediates the LPS-triggered production of various pro-inflammatory cytokines and chemokines, among which TNF-α, IL-6 and CXCL8 [38,86,87]. In addition, PI-PLC contributes to TNF-α and IL-1β production triggered by LPS in macrophages, as well as to the process of monocyte to macrophage differentiation [88]. LPS stimulation of these cells also results in the up-regulation of sPLA_2_ and cPLA_2_ activities, leading to AA release [89]. Interestingly, activation of cPLA_2_ and sustained production of eicosanoids in macrophages is also observed following systemic inflammasome triggering by flagellin, a principal component of bacterial flagella [90]. As a natural agonist of TLR5, flagellin activates the innate immune response. Elevated levels of flagellin-specific IgG were found in HIV-1-infected patients and were shown to be reduced following HAART, although not achieving the basal levels found in uninfected controls [4]. This suggests that flagellin may be an important microbial product involved in monocyte activation following microbial translocation [91].

Notably, several lines of evidence suggest that microbial translocation may play a central role in the early onset of non-AIDS co-morbidities, particularly atherosclerosis and CVD as well as neurocognitive impairment.

### 3.2. HIV-1-Associated Cardiovascular Disease

CVD encompasses a wide range of diseases that involve the heart and/or blood vessels, and is a major cause of morbidity and mortality in persons with HIV-1 infection. CVD risk is increased by approximately 1.5 to 2.0-fold among HIV-1-infected individuals compared to the general population [92]. Interestingly, HIV-1^+^ subjects have values of intima-media thickness, a subclinical marker of atherosclerosis, comparable to those of coronary artery disease patients [93]. Although HAART reduces the risk of cardiovascular complications, it does not fully restore vascular health [94]. Furthermore, some antiretroviral drugs have been implicated in the induction of lipodystrophy, which in turn promotes dyslipidemia and CVD [45]. It is still unclear whether established interventions for the general population, such as statins, are applicable to HIV-1 infected patients [92].

Several clinical studies highlighted that HIV-1^+^ individuals are particularly prone to premature atherosclerosis. Although the underlying mechanisms are not fully understood, complex interactions between traditional and HIV-1-specific risk factors jointly promote atherogenesis in individual infected with HIV-1 [45]. Chronic immune activation and inflammation are well recognized to be directly and causally associated with vascular dysfunction and accelerated atherosclerosis development in the context of HIV-1 infection acting through several mechanisms [94,95]. In recent years, accumulating evidence suggests that CVD may be a consequence of increased levels of microbial products. In this regard, LPS and flagellin were both reported to up-regulate the expression of tissue factor, which triggers the coagulation cascade on the membrane of monocytes [96]. Furthermore, LPS represents an important monocyte/macrophage activator and source of inflammatory stimuli in the setting of atherosclerosis. Indeed, high levels of LPS and sCD14 in the serum were shown to be predictive of subclinical atherosclerosis progression in HIV-1^+^ patients [97].

Monocytes/macrophages are well recognized to play an essential role in HIV-1-associated CVD pathogenesis [94]. Monocytes from infected individuals show a spontaneous overproduction of ROS [98]. An increased proportion of CD16^+^ monocytes, as well as an altered expression of adhesion molecules (ICAM-1, VCAM-1, and LFA-1) and chemokine receptors on monocytes and endothelial cells, are linked to vascular inflammation in HIV-1 infection [99]. Expression of sCD163, sCD14, fractalkine, TNFR-II, IL-6 and Lp-PLA_2_ are also increased in HIV-1-infected individuals and do not return to normal levels under HAART [93,99]. In particular, the proportion of individuals with Lp-PLA_2_ levels ≥200 ng/mL is dramatically increased among HIV-1^+^ (61%) compared to HIV-1^−^ (21%) persons, in spite of similar levels of triglycerides, LDL, and oxidized LDL between the groups [99]. Higher expression levels or increased enzymatic activity of Lp-PLA_2_ and sPLA_2_ are consistently found in HIV-1-infected patients compared to healthy controls. Interestingly, plasma Lp-PLA_2_ levels were shown to be differently affected by diverse antiretroviral regimens ([12] and references herein).

HIV-1 infection affects the three crucial events supposed to accelerate atherosclerosis progression [93]. Interestingly, each of these events is influenced by PLs, as shown in Figure 1. During the initial phase of atherosclerosis (inflammation), HIV-1 replication induces several inflammatory cytokines (i.e., TNF-α, IL-1β, IL-6, IL-12, IL-18, IFN-α), with IL-6 having a central role in up-regulating CCL2 and M-CSF, thus favoring the recruitment of monocytes and their differentiation into activated macrophages. Some HIV-1 proteins, particularly gp120 and Tat, were shown to promote the secretion of CCL2 in macrophages [75], which in turn up-regulates the expression of pro-inflammatory cytokines and adhesion molecules. In the second phase, CCL2 induces the expression of PRRs, such as CD163 and TLRs that facilitate the internalization of lipoproteins via CD36, thus promoting transformation of macrophages into foam cells. The HIV-1 protein Nef is known to favor this event by increasing CD36 expression and decreasing cholesterol efflux. In the last phase, endoplasmic reticulum (ER) stress and apoptosis of foam cells lead to plaque development. In this context, HIV-1 virus itself, as well as Nef and Tat, can induce an imbalance of Ca^2+^ disrupting ER homeostatic mechanisms, through the mediation of IP_3_ and ryanadine receptors [93].

Future investigations on the complex interplay between monocytes/macrophages, vascular endothelium and chronic inflammation may lead to improved CVD prevention and treatment in the HIV-1-infected population. In this context, PLs might be taken into consideration as potential biomarkers and/or targets for intervention, in virtue of their remarkable contribution to monocyte/macrophage-mediated inflammatory processes.

### 3.3. HIV-1-Associated Neurocognitive Disorders

HIV-1 infection is associated with CNS inflammation and neural damage and death, leading to HIV-associated neurocognitive disorder (HAND). HAND includes different and progressively more severe neurological conditions, ranging from asymptomatic neurocognitive impairment (ANI), to mild neurocognitive disorder (MND), and to HIV-associated dementia (HAD). Although HAART has been generally successful in reducing the burden of CNS disease, persistent infection of resident macrophages sustains low-level residual inflammation, thus favoring the progression of disease. Indeed, a large fraction (estimated 50%) of HIV-1^+^ patients on HAART exhibits the milder forms of brain disease, ANI and MND [100].

**Figure 3:**
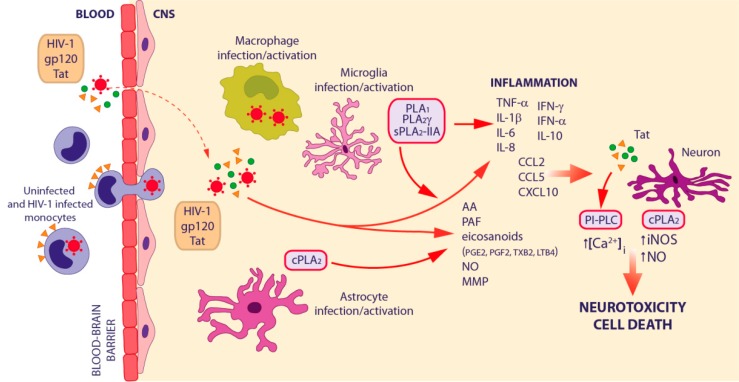
Schematic representation of concerted effects of the “direct” and the “indirect” models of HIV-1 neuropathogenesis and the involvement of different families of phospholipases. The direct effects of the virus and the secreted viral proteins (Tat, gp120) on neurons are shown, as well as the neuronal death as a consequence of the inflammatory response mounted by both infected and uninfected brain cells (macrophages, microglia, and astrocytes) against HIV-1 infection. Light purple and orange boxes represent phospholipase and HIV-1 contribution to different steps of neuropathogenesis, respectively. AA, arachidonic acid; Ca^2+^, calcium; CCL, C-C motif chemokine ligand; CNS, central nervous system; CXCL, C-X-C motif chemokine ligand; IFN, interferon; IL, interleukin; iNOS, inducible nitric oxide synthase; LT, leukotriene; MMP, matrix metalloproteinase; NO, nitric oxide; PAF, platelet-activating factor; PG, prostaglandin; TNF-α, tumor necrosis factor α; TX, thromboxane.

The neurodegeneration and neurological symptoms characterizing HAND can be explained by the concerted effects of the “direct” and the “indirect” models of HIV-1 neuropathogenesis [101] described in Figure 3, in which each PLs exerts a specific action leading to a broad effect on neuroinflammation. The first model takes into account the direct neurotoxic effects of the virus and the secreted viral proteins (Tat and gp120) on neurons through various neuronal cell surface receptors: N-Methyl-D-Aspartate Receptor (NMDAR), LDL Receptor related Protein and chemokine receptors (CCR5 and CXCR4) [101,102,103,104,105]. In particular, Tat promotes the mobilization of intracellular Ca^2+^ stores in the ER via PI-PLC-driven IP_3_ production [106]. Moreover, HIV-1 proteins can regulate the permeability of the blood–brain barrier (BBB) [107,108,109], thus increasing monocyte migration [110,111]. The “indirect” model of HIV-1 neuropathogenesis suggests that neuronal cell death is the result of the inflammatory response against HIV-1 infection mediated by both infected and uninfected brain cells [101] (Figure 3). HIV-1 enters the CNS within 1–2 weeks of systemic infection through a “Trojan Horse” mechanism, crossing the BBB inside of blood-borne monocytes that later differentiate into macrophages. After transmigration, HIV-1-infected macrophages, as well as resident microglia and astrocytes, release viral proteins (including Tat and gp120) and numerous soluble factors, such as pro-inflammatory cytokines (IL-1β, IL-6, IL-8, IFN-γ, and TNF-α), chemokines (CCL2, CCL5, and CXCL10), PAF, nitric oxide (NO), matrix metalloproteases, and AA, which jointly contribute to recruit monocytes from the periphery into the CNS, thus amplifying the cascade of inflammation and perturbing neuronal homeostasis [101]. HIV-1 infected monocytes also up-regulate several adhesion and tight junction molecules, as well as chemokine receptors, thus facilitating their own transmigration into the CNS [112]. Growing evidence is accumulating on a possible association between microbial translocation and HAND onset. In particular, it was shown that LPS triggers monocyte activation and trafficking to the brain [113]. Indeed, Ancuta and colleagues found higher circulating levels of LPS in patients with HAND, which were associated with increased plasma sCD14 and with the development of this disorder. Furthermore, sCD14 levels in cerebrospinal fluid (CSF) were associated with neurocognitive dysfunction and HIV-1-associated sensor neuropathy [4,114].

Another pathway intersecting peripheral and brain manifestations of HIV-1 infection is represented by the connection between altered monocyte/macrophage homeostasis, immune polarization and the IFN response [115]. In particular, type I IFN was reported to enhance HIV-1 and SIV expression and neuropathogenesis [116]. By recruiting IRF1 and Stat3, IFN-α induces IL-10, an important immunosuppressive Th2/M2 cytokine [117], which together with M-CSF promotes the expansion of CD14^+^/CD16^+^ monocytes [118]. This process has a deep impact in the development of CNS disease and immunosuppression. It was also reported that during acute SIV brain infection, astrocytes produce high levels of CCL2, which binds to CCR2 receptors on macrophages, leading to a selective suppression of IFN-α and a simultaneous induction of IFN-β [119].

It is generally accepted that chronic disease in individuals on effective HAART is a consequence of chronic inflammation caused by residual viremia in tissues, with additional metabolic complications of prolonged drug use [120]. Nevertheless, it is also evident that in many patients dementia is associated with the activation of macrophages/microglia rather than with viral replication in the brain [121]. Indeed, immune activation of macrophages/microglia and astrocytes was reported in patients on HAART with MND [122]. The latter cells significantly contribute to HIV-1 associated neurological disorders by releasing pro-inflammatory cytokines and modulating the CNS microenvironment. Several studies also suggest that astrocytes serve as undetected viral reservoirs in the brain and that latent infection in these cells can be easily re-activated by TNF-α or IFN-γ [123]. Direct metabolic interactions between neurons and astrocytes were reported in HAND [124,125].

Whereas macrophage/microglial and astrocytic infection are important obstacles for HIV-1 eradication, altered inflammatory pathways directly cause both immune and neurocognitive dysfunctions in patients undergoing HAART. The AA pathway is of crucial importance in affecting the production and release of other neurotoxins as well as in directly inducing cytotoxicity [126]. PLA_2_ generally removes oxidized and damaged phospholipids, thus preserving membrane structure and function; in pathological conditions, its increased activity leads to high levels of FA, lysophospholipids and their metabolites, hence contributing to neuroinflammation. AA metabolites, together with 5-hydroxyicosatetraenoic acid and PAF, affect macrophage/microglia inflammatory responses seen in a broad range of systemic degenerative and nervous system disorders [127,128,129,130]. In particular, PAF has a number of effects on innate immunity and is involved in HAND pathobiology [131], affecting monocyte/macrophage/microglia chemotaxis and recruitment [132], and neurotoxicity [133]. PAF activates both the classical NF-κB and MAPK pathways in macrophage/microglia, resulting in TNF-α, IL-1, IL-6 and IL-10 production [134,135]. TNF-α affects HIV-1 replication, thus enhancing the concentration of viral and cellular toxins as well as the production of PAF [136], which enhances HIV-1 replication [137,138]. Interestingly, PAF antagonists potently reduce neuroinflammatory responses in mouse models of HIV-1 encephalitis [139,140].

Levels of AA metabolites, including PGE_2_, PGF_2_, and TXB_2_, are increased in the CSF of HIV-1^+^ subjects with dementia and/or myelopathy with respect to infected patients without dementia and HIV-1^−^ individuals with other neurologic diseases [141]. A study employing a global metabolomic approach identified saturated and unsaturated FA and LPC among the metabolites found to be elevated in the CSF of rhesus macaques with SIV-induced CNS disease [142]. Their increase correlated with elevated expression of specific PLs, namely PLA_1_ and cPLA_2_γ. Interestingly, expression of the latter occurs in different areas of the brain together with glial activation. FFAs can induce receptor signaling through G protein-coupled receptor (GPCR)40 [143] as well as through the nuclear receptor PPARα [144], thus further altering CNS functions. Signaling through GPCRs is also triggered by LPC itself, lysophosphatidic acid [145] and PAF, which has its own GPCR and can lead to neuronal cell death [146]. LPC can also stimulate microglia to release IL-1β [147], a crucial cytokine in HAD. Moreover, higher levels of cPLA_2_α and sPLA_2_-IIA are found in brain tissues of HIV-1 transgenic rats compared to control animals [148]. Interestingly, the increased AA metabolism was associated with elevated brain markers of neuroinflammation (e.g., IL-1β and TNF-α) and with a deficit in several synaptic proteins.

Several studies demonstrated a direct effect of HIV-1 on AA or AA-metabolite production ([12] and references herein). In particular, gp120 induces AA metabolites, such as PGE_2_ and LTB_4_, in monocytes and neuroblastoma cells. In addition, HIV-1 infection of monocytes/macrophages enhances the release of AA and PGE_2_ but inhibits IL-1β production. HIV-1 also affects the AA cascade in other cells of the nervous system. High levels of LTB_4_, LTD_4_, lipoxin A4, and PAF are found in co-cultures of HIV-1–infected monocytes and astroglial cells and are associated with the induction of TNF-α and IL-1β.

Astrocyte inflammatory responses in HAND are critically regulated by cPLA_2_ signaling [149]. Indeed, human astrocyte activation by alcohol, cocaine and HIV-1 involves cPLA_2_, AA and COX_2_, thereby further inducing neuroinflammation [150]. cPLA_2_ activity is also involved in the gp120-mediated inhibition of neuronal NO synthase that allows activation of NF-κB and subsequent inducible NO synthase and IL-1β transcription in astroglial cells. The activation of iNOS is critical for the production of excessive amounts of NO, which contribute to neuronal abnormalities in HAND [151]. Overall, the results reported above suggest that the neurotoxicity linked to HIV-1–associated CNS disorders is mediated, at least in part, through cytokines and AA metabolites, produced during cell-to-cell interactions between infected brain macrophages and astrocytes. Thus, a deeper insight into the molecular and cellular mechanisms controlling the expression of these factors may help in finding new drugs to manage cognitive impairment in patients with HIV-1.

Besides the AA metabolism, also PI-PLCs are crucially involved in neuronal dysfunctions, as demonstrated by studies employing PI-PLC inhibitors [12]. Indeed, U73122 inhibits Tat-mediated production of pro-inflammatory cytokines, such as TNF-α in macrophages and astrocytic cells and IL-1β in monocytes/macrophages, as well as Tat-elicited release of norepinephrine from human and rat synaptosomes. Moreover, ET-18-OCH_3_ induces the death of HIV-1 infected microglial cells by inhibiting Akt activation and kinase activity ([12] and references herein). These data suggest a direct involvement of PI-PLC activity in macrophage/glial cell activation by HIV-1 and, thus, in the neuropathogenesis of HAND.

In the setting of HAART, some inflammatory pathways remain activated and may favor the recruitment and accumulation of M2 and/or regulatory macrophages in CNS as well as other organs, thus contributing to the neuro- and immuno-pathogenesis. Taking into account the key role of altered monocyte/macrophage homeostasis, trafficking and immune polarization [152], future therapeutic strategies for HIV-1 eradication should aim to counteract both inflammation and immune polarization.

## 4. Conclusions

Immune activation has long been considered as an important consequence of untreated HIV-1 infection and an indicator of AIDS progression, which declines but does not normalize in patients undergoing HAART, where it continues to predict disease. Persistent low levels of immune activation are associated with residual levels of viral replication during therapy, suggesting that chronic inflammation can support the replenishment of tissue HIV-1 reservoirs. Therefore, a major research agenda is to develop new treatments to counteract persistent immune activation and inflammation in virally suppressed HIV-1^+^ individuals. Thus far, the best targets for interventions remain uncertain. Monocyte and tissue macrophage activation has become increasingly recognized as a potential mediator of non-AIDS morbidity and mortality in the HAART era. Interestingly, PLs are critically involved in monocyte/macrophage functions as well as in their activation in HIV-1 infection, thus representing key players in serious non-AIDS morbidities, such as cardiovascular and neurological diseases. Then, interfering with these mechanisms may represent a strategy to attenuate the effects of inflammation and immune activation, ultimately reducing the viral reservoir, a prerequisite to remission in individuals receiving HAART. This should result in further improving quality of care and life expectancy of HIV-1-infected patients in the post-HAART era.

## Abbreviations

AA	arachidonic acid
AIDS	acquired immunodeficiency syndrome
BBB	blood–brain barrier
CCL2	C-C motif chemokine ligand 2
CNS	central nervous system
CVD	cardiovascular disease
DAG	diacylglycerol
FA	fatty acid
HAART	highly active antiretroviral therapy
HAND	HIV-associated neurocognitive disorder
HIV-1	human immunodeficiency virus type I
IP_3_	inositol 1,4,5-trisphosphate
LDL	low-density lipoprotein
LPA	lysophosphatidate
LPC	lysophosphatidylcholine
Lp-PLA_2_	lipoprotein-associated phospholipase A_2_
LPS	lipopolysaccharide
LT	leukotriene
NF-kB	nuclear factor kappa-light-chain-enhancer of activated B cells
NO	nitric oxide
PAF	platelet-activating factor
PC	phosphatidylcholine
PC-PLC	phosphatidylcholine specific phospholipase C
PG	prostaglandin
PI	phosphoinositide
PI-PLC	phosphoinositide-specific phospholipase C
PL	phospholipase
PLA_2_	phospholipase A_2_
cPLA_2_	cytosolic PLA_2_
iPLA_2_	Ca^2+^-insensitive phospholipase A_2_
sPLA_2_	secretory phospholipase A_2_
ROS	reactive oxygen species
SIV	simian immunodeficiency virus
TNF	tumor necrosis factor
